# Refractory microsatellite stable metastatic colorectal cancer with ERBB2/ERBB3 mutation may be preferred population for regorafenib plus PD-1 inhibitor therapy: a real-world study

**DOI:** 10.3389/fonc.2023.1227644

**Published:** 2023-08-23

**Authors:** Xuan Dai, Wenjun Ding, Yongshan He, Shiyong Huang, Yun Liu, Tingyu Wu

**Affiliations:** Department of Colorectal and Anal Surgery, Xinhua Hospital, Shanghai Jiao Tong University School of Medicine, Shanghai, China

**Keywords:** colorectal cancer, immune checkpoint inhibitor, microsatellite stable, regorafenib, ErbB

## Abstract

**Background:**

Microsatellite stable (MSS) colorectal cancer (CRC) has been referred to as the “cold tumor” because of almost no response to anti–programmed death-1 (PD-1) antibody. A recent REGONIVO trial showed that regorafenib plus nivolumab had an encouraging efficacy in MSS metastatic CRC (mCRC). However, only a small subset of patients may benefit from the combination therapy. We aim to evaluate the efficacy and safety data of immune checkpoint inhibitors combined with regorafenib in refractory MSS mCRC and to discover biomarkers that can effectively stratify the beneficial patient population.

**Methods:**

We retrospectively analyzed patients with MSS mCRC who received regorafenib combined with anti–PD-1 antibody therapy. The objective response rate (ORR), disease control rate (DCR), progression-free survival (PFS), overall survival (OS), and status of gene mutation were reviewed and evaluated.

**Results:**

Twenty-one patients received combination treatment. At a median treatment duration of 4 months, one patient achieved complete response, three patients achieved partial response, and two patients achieved stable disease as the best response. The ORR and DCR were 19% and 28.5% in the overall population, respectively. The median PFS was 4 months, and the median OS was 25 months. Only erbb2 receptor tyrosine kinase 2/erbb3 receptor tyrosine kinase 3 (ERBB2/ERBB3) mutation status was confirmed to be a potential predictive factor for effective treatment. In patients with ERBB2/ERBB3 mutation, ORR, DCR, and PFS exhibited significant improvements in comparison with that in wild-type patients. Grade 3 or higher treatment-related adverse events occurred in three patients (14.3%).

**Conclusions:**

Regorafenib in combination with PD-1 inhibitor provides a feasible treatment regimen for refractory MSS mCRC with tolerated toxicity. Patients with ERBB2/ERBB3 mutation may be the preferred population for this combination regimen.

## Introduction

Immune checkpoint inhibitors (ICIs) including anti–programmed death-1 (PD-1) and anti–programmed death ligand-1 (PD-L1) have demonstrated a notable efficacy in metastatic colorectal cancer (mCRC) with mismatch repair deficiency (dMMR) or high microsatellite instability (MSI-H), which were characterized by a high mutational burden and a tumor-infiltrating lymphocyte enrichment (1, 2). However, MSI-H/dMMR cancer only accounts for 2%–4% of the total mCRC cases (3). The majority of patients with CRC exhibit a microsatellite stable (MSS) or mismatch repair proficient (pMMR) status, which is known as the “cold tumor” with less mutated oncogenes and less inflamed tumor immune microenvironment, resulting in a limited efficacy of ICIs (2). The inadequate recruitment and activization of immune cells to the tumor microenvironment were considered to be fundamental mechanisms underlying the inefficacy of ICIs in MSS mCRC (4). Combination strategies to enhance the immunogenicity of the tumor microenvironment and exploit the benefit of ICIs in patients with MSS are urgently needed.

Regorafenib, a small-molecule tyrosine kinase inhibitor, has been approved for treating chemotherapy refractory mCRC (5). Meanwhile, preclinical studies have shown that regorafenib could also (a) inhibit immune-suppressive cell infiltration, (b) inhibit the expression of immunosuppressive molecules, and (c) induce reprogramming of tumor-associated macrophages (TAMs) toward an M1 phenotype, which restored the immunosuppressive tumor microenvironment and synergistically enhanced the efficacy of ICIs (6–8). A phase Ib study (REGONIVO) reported the efficacy of regorafenib plus nivolumab with an objective response rate (ORR) of 33% and a prolonged median progression-free survival (PFS) of more than 6 months in 24 patients with refractory MSS mCRC (9). Another recent phase Ib/II study of regorafenib plus toripalimab showed a less ORR of 15.2% in 33 patients with mCRC (10). These studies have shown that a subset of patients may benefit from the combination therapy. It remains a compelling clinical challenge to further identify the beneficial subset in patients with mCRC.

In 2020, we treated one patient with refractory mCRC with extensive liver and pelvis metastases using regorafenib plus nivolumab regimen for a compassionate purpose. Unexpectedly, the multiple metastases completely regressed and achieved complete response (CR) after 8 months of treatment, although her tumor genotype is MSS with a low tumor mutation burden (TMB). It has been maintained for 28 months without any recurrence or metastasis. We performed second-generation sequencing of her tumor sample and identified a simultaneous G284R mutation and amplification in ERBB3. Preclinical study has shown that ERBB2/ERBB3 mutations could promote PD-L1–mediated immune escape in gallbladder cancer (11). Because dimerized human epidermal growth factor receptor 2/human epidermal growth factor receptor 3 (HER2/HER3), expressed by ERBB2 and ERBB3, respectively (12), are the tyrosine kinase targets of regorafenib (13), we hypothesize that regorafenib may reverse immunosuppressive tumor microenvironment and synergistically enhance the efficacy of ICIs in patients with ERBB2/ERBB3 mutation or amplification.

The combination of ICIs with regorafenib may be a promising treatment strategy for patients with MSS mCRC, especially for patients with ERBB2/ERBB3 mutation. To elucidate these issues, we conducted this retrospective study to evaluate the efficacy and safety of regorafenib combined with ICIs for patients with MSS mCRC with or without ERBB2/ERBB3 mutation for compassionate usage in the real world. The impact of ERBB2/ERBB3 mutation on the efficacy of combination treatment regimen was also investigated.

## Materials and methods

### Patients

We carried out a retrospective study of patients with mCRC with MSS status receiving regorafenib and anti–PD-1 antibody in Xinhua Hospital from November 2018 to April 2023. The usage of different types of anti–PD-1 antibody, including nivolumab, toripalimab, serplulimab, and sintilimab, was determined according to the doctor’s decision. Eligibility for inclusion included the receipt of the combination of regorafenib and anti–PD-1 antibody in patients with MSS mCRC as the third- or later-line treatment for a compassionate purpose, following disease progression on standard of care therapy including Fluorouracil, Oxaliplatin, and Leucovorin (FOLFOX) and Fluorouracil, Irinotecan, and Leucovorin (FOLFIRI). Patients with prior exposure to regorafenib monotherapy were also included. The metastasis must be measurable with at least one measurable lesion according to Response Evaluation Criteria in Solid Tumors (RECIST; version1.1). The exclusion criteria included the following: (a) patients with less than two cycle of treatment, (b) patients with little information on tumor response, and (c) patients with confirmed MSI-H/dMMR status. The study was approved by the hospital ethics committee (XHEC-C-2023-069) and was carried out in accordance with the 1964 Declaration of Helsinki. A written informed consent was obtained from the patients.

### Treatment methods

Patients received oral regorafenib of 80–120 mg once a day for 21 days every 28-day cycle. The dose was reduced with a minimum dose of 80 mg for some patients to manage treatment-related toxicities. The PD-1 inhibitor (nivolumab, 3 mg/kg every 2 weeks; toripalimab, 3 mg/kg every 2 weeks; serplulimab, 3 mg/kg every 2 weeks; and sintilimab, 200 mg every 3 weeks) was intravenously administered on day 1 of oral regorafenib.

### Outcome

ORR, disease control rate (DCR), PFS, overall survival (OS), status of gene mutation, and incidence of treatment-related adverse events (TRAEs) were reviewed and evaluated.

Tumor evaluation was based on RECIST (version1.1). The response evaluation included CR, partial response (PR), stable disease (SD), and progression disease (PD). The ORR was calculated as the sum of CR and PR, whereas the DCR was the sum of CR, PR, and SD. OS was defined as the time from treatment initiation to death from any cause. PFS was defined as the time from treatment initiation to the first documented disease progression or death. TRAEs were assessed according to the Common Terminology Criteria for Adverse Events (version 5.0).

The genetic status of the patients was evaluated through post-operative pathology tests performed by the pathology department. Immunohistochemistry (IHC) staining of four kinds of MMR protein (MLH1, MSH2, MSH6, and PMS2) or polymerase chain reaction (PCR) analysis of five microsatellite markers (BAT25, BAT26, D5S346, D2S123, and D17S250) were used to determine MSI/MMR status. IHC using anti–PD-L1, anti-HER2, and anti-HER3 antibodies was performed to assess the expression status of PD-L1 and HER2/HER3. Mutation status of kirsten rat sarcoma viral oncogene homolog (KRAS), neuroblastoma ras viral oncogene homolog (NRAS), v-raf murine sarcoma viral oncogene homolog B1 (BRAF), ERBB2 and ERBB3 were determined by a PCR sequencing assay (Sanger or ARMS method). Fluorescence In Situ Hybridization (FISH) using the PathVysion HER-2 probe kit (Abbott Laboratories) was performed to assess the amplification status of HER2. All specimens in this study were reviewed by a pathologist. In addition, next-generation sequencing (NGS) analysis was conducted on the tumor samples of selected patients.

### Statistical analysis

The study was done according to the Strengthening the Reporting of Observational Studies in Epidemiology (STROBE) guidelines for observational studies (14). Difference between groups was determined by Pearson’s chi-square test or Fisher’s exact test. A multivariable logistic regression model was used to evaluate the risk of disease progression for ERBB2/ERBB3 mutation, adjusting for covariates determined *a priori* to be clinically relevant. PFS and OS were estimated by the Kaplan–Meier method and compared using the log-rank test. Statistical analysis was performed using Statistical Package for the Social Sciences (SPSS) 27 software. Statistical significance was defined at P-values <0.05.

## Result

### Baseline characteristics

A total of 21 patients with mCRC with MSS status met the study criteria and were enrolled in this study [15 men (71.4%); median age (range), 52 (32–73)] (**Table 1**). Eastern Cooperative Oncology Group Performance Status (ECOG PS) was 1 in 71.4% of patients, and ECOG PS was 0 in 28.6% of patients. Ten patients (47.6%) had synchronous metastases. Sixteen (76.2%) patients were diagnosed with left-sided primary CRC, and five (23.8%) patients were diagnosed with right-sided primary CRC. The most common metastatic sites included liver (66.7%), lung (33.3%), peritoneum (28.6%), and lymph node (19%). All patients had received ≥3 previous lines of chemotherapy, including anti-Vascular Endothelial Growth Factor (VEGF) treatment administered to 95.2% of patients. Nine (42.9%) patients had previously received regorafenib treatment, and all of them experienced disease progression before the combination treatment. The MSS/pMMR status was confirmed in 21 patients (100%). Eight patients (38.1%) had KRAS or NRAS mutant status, and three patients (14.3%) had BRAF V600E mutations. Five patients (23.8%) had ERBB2/ERBB3 mutation or amplification status (two patients with ERBB2 mutation, two patients with ERBB2 amplification, and one patient with synchronous ERBB3 mutation and amplification), whereas 16 patients (76.2%) were wild type. The types of anti–PD-1 antibody included nivolumab (61.9%), toripalimab (19%), serplulimab (9.5%), and sintilimab (9.5%). Among them, toripalimab, serplulimab, and sintilimab were Chinese domestic ICIs.

**Table 1:** Baseline demographic and clinical characteristics of 21 patients with MSS mCRC.

Characteristics	Total no. (n = 21)
Median age, years (range)	52 (32–73)
Age	
≥ 60	9 (42.8%)
< 60	12 (57.2%)
Gender	
Male	15 (71.4%)
Female	6 (28.6%)
ECOG performance status	
= 1	15 (71.4%)
= 0	6 (28.6%)
Primary tumor location	
Cecum and ascending colon	4 (19%)
Transverse colon	1 (4.7%)
Descending colon	4 (19%)
Sigmoid colon	5 (23.8%)
Rectum	7 (33.3%)
Synchronous metastases	10 (47.6%)
Site of metastases	
Liver	14 (66.7%)
Lung	7 (33.3%)
Lymph node	4 (19%)
Peritoneum	6 (28.6%)
Bone	3 (14.2%)
Ovary	2 (9.5%)
Prior systemic treatment lines	
3	2 (9.5%)
4	15 (71.4%)
5	3 (14.2%)
Prior systemic treatment regimens	
Fluoropyrimidines	21 (100%)
Oxaliplatin	21 (100%)
Irinotecan	21 (100%)
Anti-EGFR treatment	9 (42.9%)
Anti-VEGF treatment	20 (95.2%)
Regorafenib	9 (42.9%)
Baseline NLR	
≥ 1.5	16 (76.2%)
< 1.5	5 (23.8%)
Anti–PD-1 antibodies	
Nivolumab	13 (61.9%)
Toripalimab	4 (19%)
Serplulimab	2 (9.5%)
Sintilimab	2 (9.5%)
KRAS/NRAS mutation	8 (38.1%)
BRAF mutation	3 (14.3%)
ERBB2/ERBB3 mutation or amplification	
Yes	5 (23.8%)
No	16 (76.2%)

ECOG, Eastern Cooperative Oncology Group; NLR, neutrophil–lymphocyte ratio.

### Efficacy

**Figure 1:**
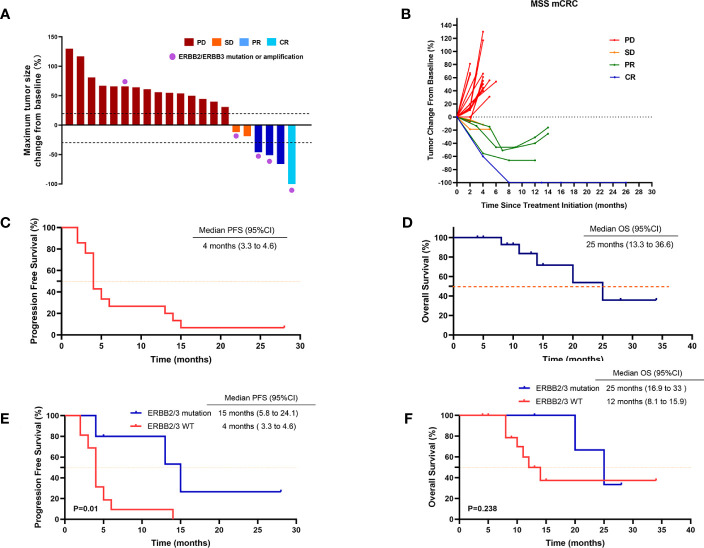
Tumor response in patients treated with regorafenib and anti–PD-1 antibody. **(A)** Waterfall plot of maximum percent change in tumor size from baseline as measured according to RECIST 1.1 in 21 evaluated patients. **(B)** Spider plot of longitudinal change in individual tumor burden over time in RECIST percentage from baseline. **(C)** Kaplan–Meier plot of progression-free survival (PFS) in 21 patients. **(D)** Kaplan–Meier plot of overall survival (OS) in 21 patients. **(E)** PFS in patients with or without HER2/HER3 mutation. **(F)** OS in patients with or without HER2/HER3 mutation. Data cutoff date for survival results was 1 May 2023. P < 0.05 was considered statistically significant.

The median treatment duration was 4 months (range, 2–28 months). One patient (4.8%) achieved CR, three patients (14.3%) achieved PR, and two patients (9.5%) achieved SD as the best response (**Table 2**; **Figures 1A, B**). The ORR and DCR were 19% (four of the 21 patients) and 28.5% (six of the 21 patients) in the overall population, respectively. Three patients had ongoing responses at the time of analysis, including one patient with CR for 28 months. All 21 patients were evaluable for PFS and OS. The median PFS was 4 months (95% CI, 3.3–4.6) (**Table 2**; **Figure 1C**), and the median OS was 25 months (95% CI, 13.3–36.6) (**Table 2**; **Figure 1D**).

**Table 2:** Anti-tumor efficacy of regorafenib plus PD-1 inhibitor in 21 patients with MSS mCRC.

	Patients with MSS mCRC, no. (%)			
Parameter	Total (n = 21)	ERBB2/ERBB3 mutation (n = 5)	ERBB2/ERBB3 wild type (n = 16)	*P ^a^*
Best response				0.011*
CR	1 (4.8%)	1 (20%)	0 (0%)	
PR	3 (14.3%)	2 (40%)	1 (6.2%)	
SD	2 (9.5%)	1 (20%)	1 (6.2%)	
PD	15 (71.4%)	1 (20%)	14 (87.5%)	
ORR (CR + PR)	4/21(19%)	3/5 (60%)	1/16 (6.2%)	0.028*
DCR (CR + PR + SD)	6/21 (28.5%)	4/5 (80%)	2/16 (12.5%)	0.011*
PFS, median month (95% CI)	4 (3.3–4.6)	15 (5.8–24.1)	4 (3.3–4.6)	0.01*
OS, median month (95% CI)	25 (13.3–36.6)	25 (16.9–33)	12 (8.1–15.9)	0.238

ORR, objective response rate; DCR, disease control rate; CR, complete response; PR, partial response; SD, stable disease; PD, progression disease; PFS, progression-free survival; OS, overall survival.

a P-values indicate differences between the HER2/HER3 mutation group and the HER2/HER3 wild-type group. Fisher’s exact test was used to in ORR and DCR comparison. Log-rank test was used in PFS and OS comparison. P < 0.05 was considered statistically significant. *P < 0.05.

### Subgroup analysis of predictive factors

We also performed univariate analysis to evaluate the predictive value of clinicopathologic factors for DCR, including age (≥ 60 *vs*. < 60), ECOG (1 *vs*. 0), primary tumor site (left colorectum *vs*. right colon), synchronous metastases (yes *vs*. no), liver metastasis (with *vs*. without), lung metastasis (with *vs*. without), previous regorafenib treatment (yes *vs*. no), baseline neutrophil–lymphocyte ratio (NLR) (≥ 1.5 *vs*. < 1.5), KRAS status (wide type *vs*. mutant), BRAF status (wide type *vs*. mutant), and ERBB2/ERBB3 status (wide type *vs*. mutant) (**Table 3**). Only ERBB2/ERBB3 mutation status was confirmed to be a potential predictive factor and associated with the increased risk of disease control [OR, 28 (95% CI, 1.9–394.4); p = 0.014] (**Table 3**). After adjusting for ECOG PS, Rat Sarcoma (RAS) mutation status, BRAF mutation status, and liver metastasis, the increased disease control risk for ERBB2/ERBB3 mutation remained significant [adjusted Odds Ratio (aOR), 54.8 (95% CI, 1.2–2497.3); p = 0.04).

**Table 3:** Analysis of risk factors for disease progression and disease control.

	Disease control *vs*. disease progression		
Variable	DCR (CR + PR + SD) (n = 6)	PD (n = 15)	*P* ^a^
Age			
≥ 60	3 (50%)	6 (66.7%)	1.00
< 60	3 (50%)	9 (75%)	
ECOG			
= 1	4 (66.7%)	11 (73.3%)	1.00
= 0	2 (33.3%)	4 (26.7%)	
Site of primary tumor			
Left	6 (100%)	10 (66.7%)	0.262
Right	0 (0%)	5 (33.3%)	
Synchronous metastases			
Yes	4 (66.7%)	6 (40%)	0.361
No	2 (33.3%)	9 (60%)	
Liver metastases			
Yes	5 (83.3%)	9 (60%)	0.613
No	1 (16.7%)	6 (40%)	
Lung metastases			
Yes	1 (16.7%)	7 (46.7%)	0.336
No	5 (83.3%)	8 (53.3%)	
Previous regorafenib			
Yes	3 (50%)	6 (40%)	1.00
No	3 (50%)	9 (60%)	
Baseline NLR			
≥ 1.5	4 (66.7%)	12 (80%)	0.598
< 1.5	2 (33.3%)	3 (20%)	
KRAS/NRAS mutation			
Yes	1 (16.7%)	7 (46.7%)	0.336
No	5 (83.3%)	8 (53.3%)	
BRAF mutation			
Yes	1 (16.7%)	2 (13.3%)	1.00
No	5 (83.3%)	13 (86.7%)	
ERBB2/ERBB3 mutation			
Yes	4 (66.7%)	1 (6.7%)	0.011*
No	2 (33.3%)	14 (93.3%)	

DCR, disease control rate; CR, complete response; PR, partial response; SD, stable disease; PD, progression disease; ECOG, Eastern Cooperative Oncology Group Performance Status; NLR, neutrophil–lymphocyte ratio.

a P-values indicate differences between the disease control group and the disease progression group patients. Fisher’s exact test was used for comparison between groups. P < 0.05 was considered statistically significant. *P < 0.05.

Moreover, the ORR and DCR were significantly higher in patients with ERBB2/ERBB3 mutation (60% and 80%, respectively), compared with that in wild-type patients (6.2% and 12.5%, respectively) (**Table 2**). Median PFS was 15 months in patients with ERBB2/ERBB3 mutation and 4 months in wild-type patients (p = 0.01) (**Table 2**; **Figure 1E**). However, there were no significant differences observed in the OS between the groups (p = 0.238) (**Table 2**; **Figure 1F**).

### Safety

Combination treatment was well tolerated, with no grade 4 or above toxicities being recorded while on treatment (**Table 4**). Of the 21 patients, 76% of patients had grade 1–2 TRAE and 14.3% of patients had grade 3 TRAE. The most common grade 1–2 TEAEs included hypertension (28.5%), decreased appetite (28.5%), fatigue (19%), diarrhea (19%), transaminase elevation (19%), and hand–foot skin reaction (14.2%). Two patients (9.5%) had grade 3 transaminase elevation, and one patient (4.7%) had grade 3 myocardial enzyme elevation.

**Table 4:** Adverse events of combination treatment of regorafenib and anti–PD-1 antibodies.

	Patients (n = 21)		
Adverse events	Any grade	Grades 1–2	Grade 3
All	16 (76%)	16 (76%)	3 (14.3%)
Fatigue	4 (19%)	4 (19%)	0
Hand–foot skin reaction	3 (14.2%)	3 (14.2%)	0
Hypertension	6 (28.5%)	6 (28.5%)	0
Decreased appetite	6 (28.5%)	6 (28.5%)	0
Diarrhea	4 (19%)	4 (19%)	0
Transaminase elevation	4 (19%)	2 (9.5%)	2 (9.5%)
Myocardial enzyme elevation	1 (4.7%)	0	1 (4.7%)
Rash	2 (9.5%)	2 (9.5%)	0
Pneumonia	1 (4.7%)	1 (4.7%)	0
Vomiting	2 (9.5%)	2 (9.5%)	0
Fever	1 (4.7%)	1 (4.7%)	0

### A case report of complete response

**Figure 2:**
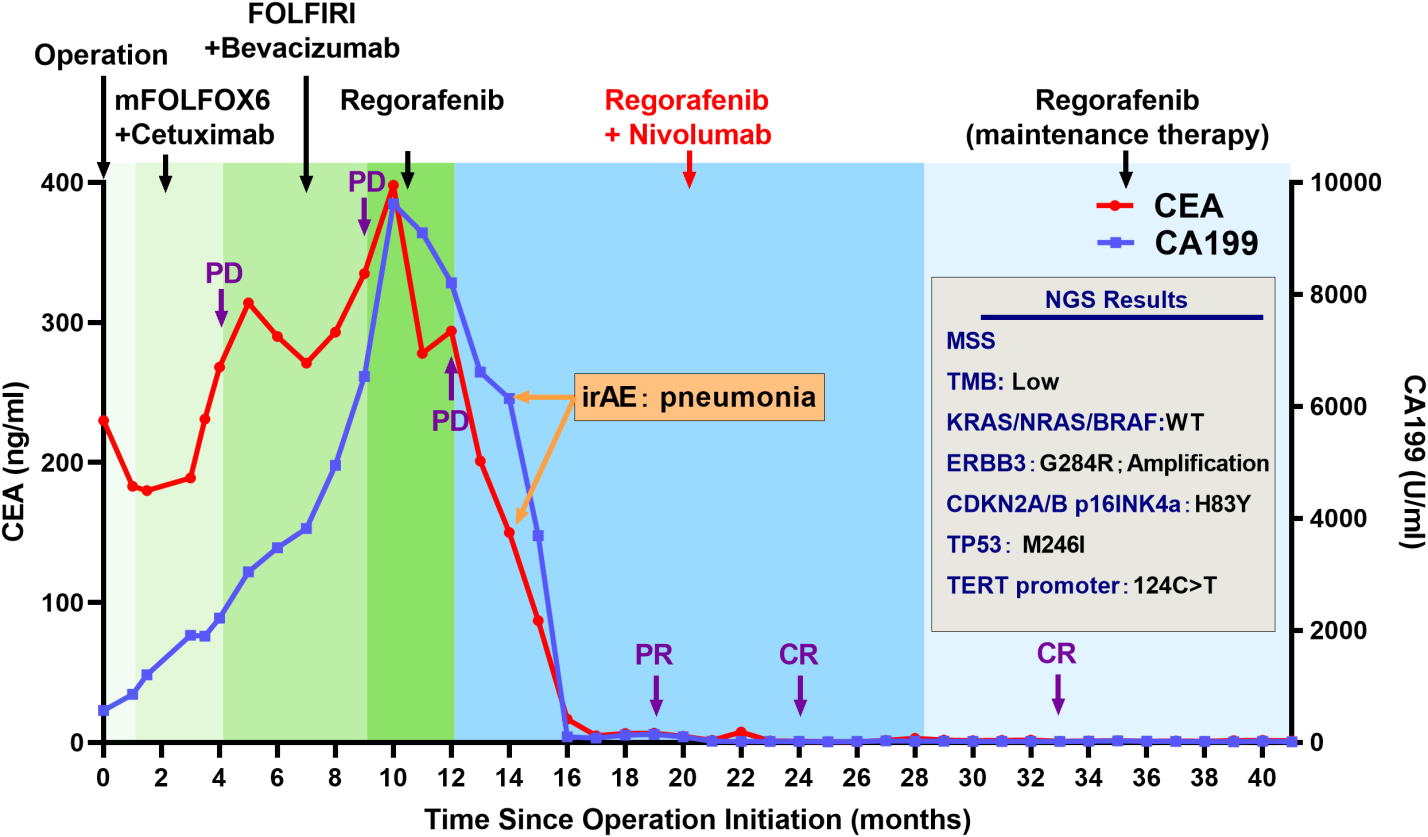
Clinical history and tumor marker levels of the patient with complete response during treatment. Treatment and molecular testing are indicated in the figure.

**Figure 3:**
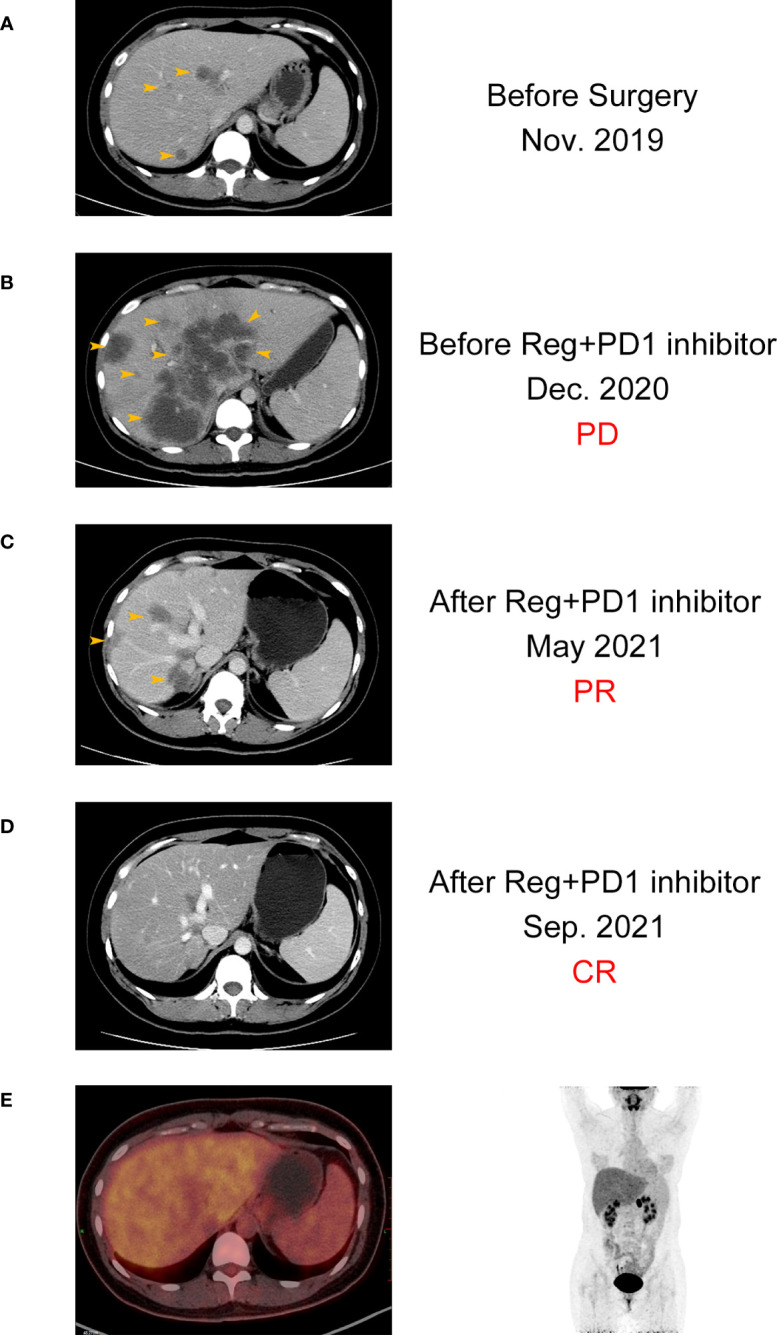
CT images of the patient with complete response before **(A, B)** and after **(C, D)** the combination treatment. **(E)** PET-CT images of the patient after the combination treatment.

Here, we present the case of a 32-year-old female patient diagnosed with a refractory metastatic colon cancer with a short expected survival time after progression of the third-line therapy. She was first diagnosed with sigmoid colon cancer in November 2019 with multiple liver and pelvic metastasis and hemorrhagic ascites. Because of the incomplete intestinal obstruction, she underwent colectomy with no metastasis excision on 11 November 2019. The post-operative pathology identified adenocarcinoma (pT4N2cM1) with KRAS/NRAS/BRAF wild-type MSS status in her tumor. Unfortunately, after five cycles of mFOLFOX6 plus cetuximab and FOLFIRI plus bevacizumab, respectively, she was found to have ongoing disease progression with increased Carcinoembryonic Antigen (CEA) (from 230 ng/mL to 398 ng/mL), CA199 (from 570 U/mL to 9,620 U/mL), and liver metastatic lesion size (**Figures 2**, **3**). After three cycles of regorafenib [120 mg daily (dq)] monotherapy used as the third-line treatment, minor decrease of CEA (from 398 ng/mL to 294 ng/mL) and CA199 (from 9,620 U/mL to 8,210 U/mL) were observed (**Figure 2**). However, CT scanning revealed a severe disease progression in liver in December 2020 (**Figure 3**).

The patient was subsequently treated with nivolumab [200 mg every two weeks (q2w)] plus regorafenib (120 mg qd) from December 2020 for a compassionate purpose (**Figure 2**). However, after 2 months of combination treatment, the patient developed immune-associated pneumonia (grade 2) with cough and fever. The patient successfully recovered from pneumonia after discontinuing the treatment for one month and received dexamethasone treatment for 1 week and, subsequently, opted to undergo re-challenge with the nivolumab (180 mg q2w) plus regorafenib (80 mg qd) regimen. Unexpectedly, the multiple liver and pelvic metastases exhibited a rapid regression and achieved a CR with abrupt decline of CEA (from 398 ng/mL to 3 ng/mL) and CA199 (from 8,210 U/mL to 35 U/mL) following an 8-month course of treatment. After combination treatment for 16 months, the patient received regorafenib (80 mg qd) for the maintenance therapy and resumed her regular occupational and daily activities. Until May 2023, this CR has been sustained for a duration of 28 months, without any evidence of recurrence or metastasis.

NGS using the patient’s tumor tissue sample was tested and identified the ERBB3 G284R mutation and amplification as major oncogenic molecular alteration. Moreover, mutation of CDKN2A/B p16INK4a (H83Y), TP53 (M246I), and TERT promter (124C>T) and low TMB were also detected (**Figure 2**).

## Discussion

Although ICIs have demonstrated a remarkable efficacy in patients with MSI-H status, which only account for 2%–4% of the total mCRC cases (3). MSS-type CRC, characterized by a low tumor mutational burden and a negligible immune cell infiltration, has been referred to as the “cold tumor” because of almost no response to immunotherapy (4). Current research studies are actively investigating the viability of combination strategies as a means of converting MSS “cold tumor” into an immune-responsive “hot tumor.” Although the combination of ICIs with chemotherapy, bevacizumab, cetuximab, and Mitogen-Activated Protein Kinase Kinase (MEK) inhibitor has been investigated in some clinical trials, these studies failed to show a significant improvement in ORR, PFS, or OS with this combination (15–18).

Immunosuppressive cells, including regulatory T cells (Tregs), and TAMs, are present within the tumor microenvironment of patients with MSS colorectal cancer. These cells can effectively suppress the activity of T cells. Preclinical research studies had demonstrated that the multi-kinase inhibitor regorafenib can alleviate the immunosuppressive effects of Tregs and TAMs on T cells by inhibiting Colony Stimulating Factor 1 Receptor (CSF1R) and Vascular Endothelial Growth Factor Receptor (VEGFR) (6–8). This mechanism can be utilized to overcome ICI resistance in MSS CRC.

A recent Japanese trial, the REGONIVO study, reported an ORR of 36% and PFS of 7.9 months in 25 patients with MSS mCRC (9). In the North American REGONIVO trial, the ORR of regorafenib combined with nivolumab was 7% among patients with MSS mCRC. The PFS and OS were 1.8 and 11.9 months, respectively, both of which were inferior to those observed in Japanese REGONIVO study (19). In contrast to the findings of the REGONIVO study, a recent retrospective study of 18 patients with MSS mCRC revealed a poor clinical activity of regorafenib combined with nivolumab or pembrolizumab. Only 31% of the DCR was observed, and no patients demonstrated an objective response (20). The authors of the study suggest that the clinical use of this combination should be avoided in patients with MSS mCRC, particularly those with liver metastases.

Our present study evaluated the efficacy of regorafenib and anti–PD-1 antibodies as the third-line or above therapy in 21 patients with refractory MSS mCRC. In general, our treatment regimen demonstrated a certain degree of therapeutic efficacy in patients. The overall ORR and DCR reached 19% and 28.5%, respectively, with one patient with CR, three patients with PR, and two patients with SD being observed, although our response rates were lower than that in the Japanese REGONIVO study. Furthermore, the median PFS and OS were found to be 4 and 25 months, respectively. Notably, among patients with previously treated mCRC who had undergone regorafenib treatment and experienced disease progression, the median PFS and OS were 2 and 7 months, respectively (21, 22). Our study outcomes were found to be more favorable compared with that in the conventional treatment. Therefore, combination therapy could be a feasible treatment option for refractory MSS colorectal cancer.

Despite the promising results of the combination therapy, a considerable portion of MSS CRC (71.5%) have exhibited disease progression. Therefore, it is crucial to further investigate biomarkers that can effectively stratify the patient population and enhance the survival benefit. In the subgroup analysis of predictive factors for DCR, the clinical benefit of the treatment was correlated with ERBB2/ERBB3 status. Patients with ERBB2/ERBB3 mutation responded well to this combination regimen (ORR, 60% *vs*. 6.2%; p = 0.028). The median PFS in patients with ERBB2/ERBB3 mutation was significantly better than that in wild-type patients (15 months *vs*. 4 months; p = 0.022). In the only CR patient, we found that the patient had both ERBB3 G284R mutation and ERBB3 amplification.

HER2 and HER3, expressed by ERBB2 and ERBB3, respectively, are tyrosine kinase receptors that form heterodimers in cell membrane (12). Mutation of ERBB2/ERBB3 results in an abnormal activation of ERBB signaling pathway and promotes tumor proliferation and metastasis, which can be inhibited by regorafenib (13). Whole-exome sequencing identified ERBB2 and ERBB3 mutation (including short-variant mutation and copy number amplification) at a frequency of 6.5%–11.5% in CRC, and patients with ERBB2/ERBB3 mutations exhibited poorer prognoses (23, 24). HER2/HER3 may also serve as an attractive therapeutic target for the treatment of CRC with ERBB2/ERBB3 mutation (25, 26). Preclinical studies have revealed that genomic ERBB2/ERBB3 mutations promote PD-L1–mediated immune escape in gallbladder cancer through inhibiting the ability of tumor-reactive T cells and attenuating the release of Interferon (IFN-γ) and Interleukin-2 (IL-2) (11). Combination treatment with an ERBB signaling pathway inhibitor and anti–PD-1 antibody reversed these immunosuppressive effects and revealed promising therapeutic activities (11).

Previous reports have identified ERBB2 and ERBB3 alteration at a frequency of 6.5%–11.5% in CRC (23, 24), whereas 23.8% of patients with mCRC exhibited alterations in ERBB2/ERBB3 in our study. This study is a retrospective analysis in the real-world setting, where treatment regimens were based on the actual patients’ conditions. For patients with refractory MSS mCRC with ERBB2/ERBB3 alterations, we are inclined to use either single-agent regorafenib or combination therapies, as mutated ERBB2/ERBB3 is one of the targets of regorafenib, which leads to a higher incidence of ERBB2/ERBB3 alterations in our study population. However, the elevated incidence of ERBB2/ERBB3 alterations does not impact the analysis of results. As reported by public databases (27, 28), alterations in ERBB2 and ERBB3 in patients with CRC are not correlated with DFS and OS. Higher alteration rates of ERBB2/ERBB3 do not predict a better prognosis or efficacy in our study.

The tolerability of the combination therapy’s toxicity profile was comparable to that of the previous studies, and the incidence of TRAEs was similar to conventional treatments such as regorafenib monotherapy (9, 19, 20). Notably, the only patient in this study who achieved CR developed immune-related pneumonia (ir-pneumonia, grade 2) after 2 months of treatment, possibly due to overactive immune response. After the discontinuation of treatment for 1 month and the administration of dexamethasone, the patient recovered. During the onset of pneumonia, there was a significant decrease in CEA and CA199, and re-challenge treatment led to the achievement of CR. Hence, irTRAEs may predict a better antitumor effect of PD-1 therapy, and a careful consideration should be given to balancing the risk of irTRAEs with the potential efficacy benefits.

This study has several limitations. First, this is a retrospective study with a small sample size. Thus, any efficacy analysis was preliminary, and the role of ERBB mutation as a potential biomarker could not be fully evaluated. Prospective validations of this strategy in large cohorts are required. We are currently conducting a phase II prospective study to evaluate the potential efficacy of regorafenib plus toripalimab in patients with MSS mCRC with ERBB2/ERBB3 mutations. Second, this is a real-word study including four different anti–PD-1 antibodies used in this study. The doses of regorafenib and anti–PD-1 antibodies were not uniform between patients, which would further increase the heterogeneity of this study.

On the basis of our findings, we speculate that ERBB2/ERBB3 mutations lead to immune escape in patients with MSS, and regorafenib can reactivate the tumor microenvironment by targeting the ERBB pathway, transforming “cold tumor” into “hot tumor,” thereby synergistically enhancing the therapeutic effects of anti–PD-1 antibodies. In conclusion, we found that regorafenib, in combination with PD-1 inhibitor, provides a feasible treatment regimen for chemotherapy refractory MSS mCRC with tolerated toxicity. Patients with ERBB2/ERBB3 mutation may be sensitive to this combination regimen.

## Data availability statement

The original contributions presented in the study are included in the article/supplementary material. Further inquiries can be directed to the corresponding authors.

## Ethics statement

The studies involving humans were approved by xinhua hospital ethics committee. The studies were conducted in accordance with the local legislation and institutional requirements. The participants provided their written informed consent to participate in this study. Written informed consent was obtained from the individual(s) for the publication of any potentially identifiable images or data included in this article.

## Author contributions

TW and YL: conceptualization, data curation, funding acquisition, supervision, and writing. XD and WD: data collection, data analysis, and methodology. SH and YH: data collection. All authors contributed to the article and approved the submitted version.
